# A spatial-temporal statistical analysis of health seasonality: explaining HFMD infections within a children population along the Vietnamese south central coast

**DOI:** 10.1186/s12889-019-7281-4

**Published:** 2019-07-11

**Authors:** Phuong N. Truong, Thuong Vu Nguyen, Thao Thi Thanh Nguyen, Alfred Stein

**Affiliations:** 1 Faculty of Geo-Information Science and Earth Observation, University of Twente, Enschede, The Netherlands; 2 Pasteur Institute, Ho Chi Minh City, Viet Nam

**Keywords:** Health seasonality, NTDs, Childhood HFMD, STAR, Fourier analysis, Disease, dynamics, health geography, small area analysis.

## Abstract

**Background:**

Various neglected tropical diseases show spatially changing seasonality at small areas. This phenomenon has received little scientific attention so far. Our study contributes to advancing the understanding of its drivers. This study focuses on the effects of the seasonality of increasing social contacts on the incidence proportions at multiple district level of the childhood hand-foot-mouth disease in Da Nang city, Viet Nam from 2012 to 2016.

**Methods:**

We decomposed the nonstationary time series of the incidence proportions for the nine spatial-temporal (S-T) strata in the study area, where S indicates the spatial and T the temporal stratum. The long-term trends and the seasonality are presented by the Fourier series. To study the effects of the monthly average ambient temperature and the period of preschooling, we developed a spatial-temporal autoregressive model.

**Results:**

Seasonality of childhood hand-foot-mouth disease incidence proportions shows two peaks in all spatial strata annually: large peaks synchronously in April and small ones asynchronously during the preschooling period. The peaks of the average temperature are asynchronous with the seasonal peaks of the childhood hand-foot-mouth disease incidence proportions in the period between January and May, with the negative values of the regression coefficients for all spatial strata, respectively: ${\beta}_{{\mathrm{T}}_11}^{S_1}=-0.18\pm 0.07;{\beta}_{{\mathrm{T}}_11}^{S_2}=-0.25\pm 0.09;{\beta}_{{\mathrm{T}}_11}^{S_3}=-0.14\pm 0.05$. The increasingly cumulative preschooling period and the seasonal component of the incidence proportions are negatively correlated in the period between August and December, with the negative values of the regression coefficients for all temporal strata, respectively: ${\beta}_{{\mathrm{T}}_32}^{S_1}=-0.40\pm 0.01;{\beta}_{{\mathrm{T}}_32}^{S_2}=-0.29\pm 0.00;{\beta}_{{\mathrm{T}}_32}^{S_3}=-0.25\pm 0.01$.

**Conclusions:**

The study shows that social contact amongst children under five years of age is the important driving factor of the dynamics of the childhood hand-foot-mouth disease outbreaks in the study area. The preschooling season when children’s contact with each other increases stimulates the geographical variation of the seasonality of childhood hand-foot-mouth disease infections at small areas in the study area.

## Background

Neglected tropical diseases (NTDs) have shown evidently seasonal patterns with various timings, amplitudes and types [1–3]. NTDs remain socio-economic burdens in developing countries, especially in low-income communities in Asia and Africa. The considerable seasonality of these NTDs is affected by seasonal changes of both environmental and social risk factors [4, 5]. Understanding the regularity or irregularity of NTDs’ outbreaks is necessary to obtain optimal control over the disease spread [6], to reduce their burdens and to reach the UN sustainable development goals for health [7].

Amongst others, the infections of *Coxsackievirus A16* (CA16) and human *Enterovirus 71* (EV71) that mainly cause hand-foot-mouth disease (HFMD) in children under 5 years of age (U5s) have been recognized as an emerging public health problem in North East and South East Asian countries [8, 9]. So far, studies of the seasonal variations of childhood HFMD infections have mainly focused on climatic driving factors such as temperature, humidity and rainfall [9]. The effects of seasonally changing social contacts on the dynamics of childhood HFMD for small areas have been overlooked, even though HFMD viruses are mainly transmitted by directly physical contacts between infected and non-infected children [10]. Additional research has been done for northeastern Asian countries [11–13], whereas some studies were also done for southeastern Asian regions [14, 15]. Despite the increasing application of mathematical models to understand the epidemiology of infectious diseases, spatial-temporal statistical methods (STS) have not yet been well recognized and leveraged for studying seasonality of NTDs, i.e. to quantitatively explain the spatial-temporal dynamics of the disease outbreaks [16], especially for small geographical areas.

In this research, we study the seasonality of childhood HFMD in Viet Nam and its geographical variations at small areas as affected by seasonally changing weather and the social contacts. The study of Horby et al. in Viet Nam in 2011 [17] shows that the physical contact amongst U5s themselves is the most intensive, especially during the period at preschool. Hence, the social contact amongst U5s is measured by the annually cumulated period of time they spend at preschool since their first day at preschool. We distinguish between the trend and the seasonality. The trend establishes the long-term changing pattern of the mean level, whereas the seasonality represents yearly periodic variations. We propose a two stage STS analysis to extract the seasonal patterns and to identify the driving factors of the incidences of childhood HFMD infections in Da Nang city, Viet Nam as an application. In so doing, we aimed to identify and understand the effects of seasonally changing social contacts of U5s on the dynamics of HFMD outbreaks at multiple district level.

## Methods

### Study area and data

**Fig. 1:**
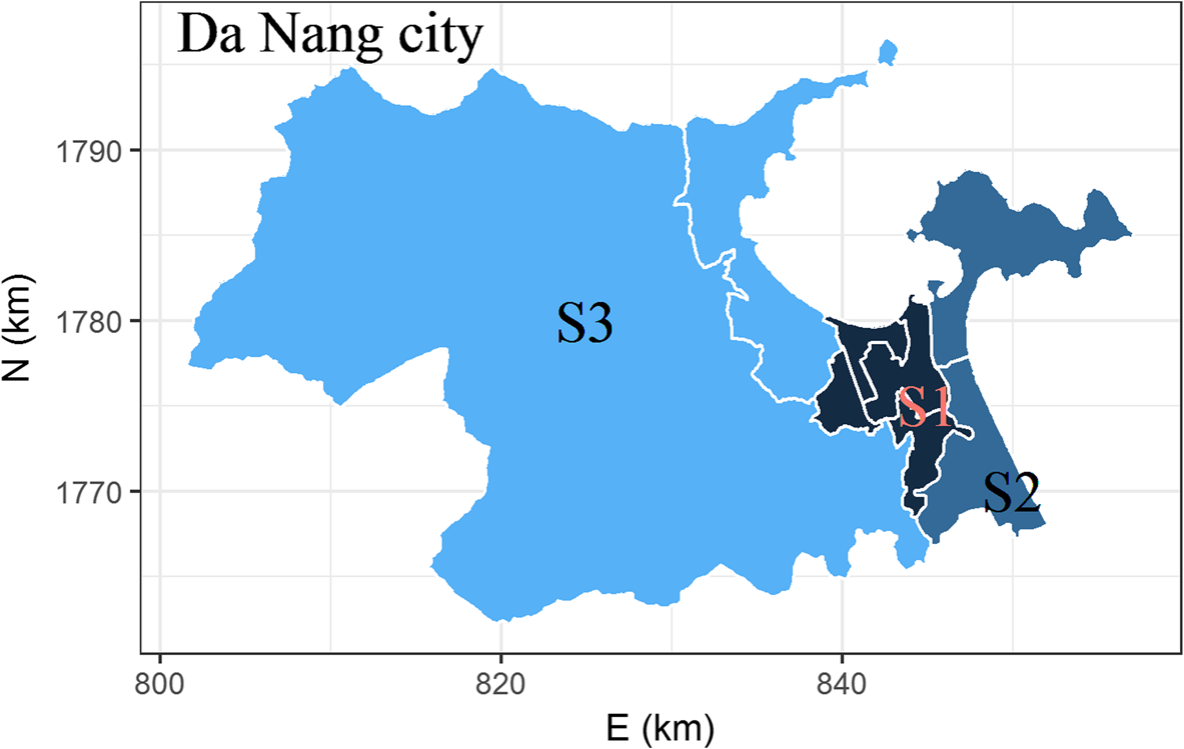
Spatial strata in the mainland of Da Nang city, Viet Nam: S_1_ is the most densely populated districts, S_3_ has the lowest population density as compared to S_1_ and S_2_

Da Nang city (Fig. 1), the biggest city on the south central coast of Viet Nam has an area of 1,285 km^2^ and a population of more than 1 million people in 2016 [18]. Approximately 8% of Da Nang city’s population consists of U5s [18]. According to the guideline from the Vietnamese Ministry of Health on diagnosis and treatment of HFMD, a confirmed HFMD case is defined when a child has a positive RT-PCR assay for CA16 or EV71. Weekly counts of new cases, i.e. the incidences from seven mainland districts of the city from 2012 to 2016 were obtained from the published reports of Da Nang city Preventive medicine center (https://ksbtdanang.vn/). Because these weekly counts have many small number of cases (< 5 cases per week), statistics based upon them are not reliable. To avoid this small number problem, the monthly count of new cases that is the sum of the weekly counts of new cases from this month was analyzed in this study. Note that the first and last weeks of the months can include 1 to 3 days of the previous or the next months. The demographical data were obtained from the demographic statistics of Da Nang city. The monthly population of U5s was interpolated from the yearly data with the assumption of a constant monthly growth rate. The monthly meteorological data from 2012 to 2016 were obtained from the Viet Nam institute of meteorology, hydrology and climate change (http://www.imh.ac.vn).

### Spatial-temporal heterogeneity analysis

To analyze the spatial-temporal heterogeneity and identify the predominant risk factors of HFMD, we compared the *q*-statistic of the nine spatial-temporal stratification to that of without stratification: $q=1-\frac{\sum \limits_{h=1}^H{N}_h{\sigma}_h^2}{N{\sigma}^2}$ [19]; where: *N* is the total number of HFMD cases in the study area and *σ*^*2*^ is the corresponding total variance, *N*_*h*_ and ${\sigma}_h^2$ are those of various spatial-temporal strata that are considered in the statistical test, *H* is the total number of these strata. The *q*-statistic for heterogeneity analysis regarding covariates was provided by the geodetector library of R [19, 20].

### Spatial-temporal stratification

To evaluate the effects of U5s as the population at risk on the magnitude of the seasonality of the childhood HFMD incidences, we stratified the districts by the density of U5s. This classification resulted into three spatial strata (Fig. 1): S_1_ is an urban stratum with the highest density of U5s (> 500 per km^2^), S_3_ is the rural stratum having the lowest density (< 100 per km^2^) and S_2_ is located in between.

We transformed the monthly counts of HFMD incidences to the monthly HFMD incidence proportions in order to correct the effects of the internal heterogeneity of the population at risk within the spatial strata. Let *i* = 1:12 index the months, *j* = 1:5 the years, *d* = 1:7 the districts and *s* = 1:3 the spatial strata. ${IR}_{ij}^s$ is used to denote the incidence proportion at spatial stratum *s* in month *i* of year *j*. To account for unequal numbers of days in months, the ${IR}_{ij}^s$ were adjusted as: ${IR}_{ij}^s=\frac{365.25\times \sum \limits_s{I}_{ij}^d}{12\times {D}_i\times \sum \limits_s{N}_{ij}^d\times \mathrm{1,000}}$ [21], where ${I}_{ij}^d$ is the monthly incidence proportion, ${N}_{ij}^d$ is the population of U5s, *D*_*i*_ is the number of days per month, $\sum \limits_s{I}_{ij}^d$ is the sum of HFMD incidences of the districts and $\sum \limits_s{N}_{ij}^d$ is the total population of U5s belonging to the stratum *s*. This adjustment removes the false variations of ${IR}_{ij}^s$ attributed to the variations of the at-risk populations and the length of time unit, partly due to the use of the weekly reported data. ${IR}_{ij}^s$ therefore equals the number of infected U5s per 1,000 U5s at risk per month, provided that removing the infected children from the population at risk only has a marginal correction effect.

### Modelling seasonality

The seasonality of ${IR}_{ij}^s$ was diagnosed by using the seasonal index (SI). The original value of ${IR}_{ij}^s$ for each stratum *s* in month *i* in year *j* was represented as the percentage of the mean value of the ${IR}_{ij}^s$ for stratum *s* in year *j*. SI of stratum *s* in month *i* equals the median of the percentages from all 5 years: ${SI}_i^s= median\left(\frac{IR_{ij}^s}{12^{-1}\sum \limits_{i=1}^{12}{IR}_{ij}^s}\times 100,j=1:5\right)$ [22]. The median allows for the inclusion of the effects of extreme values over the years. The standard error of SI was derived from the bootstrap confident interval assuming a normal distribution. A value of ${SI}_i^s$ > 100% indicates that the incidence proportion is above the yearly average incidence proportion and vice versa.

For the analysis of the dynamics, we applied to ${IR}_{ij}^s$ an additive conceptual model with three main components: a trend (*μ*), a seasonality (*ξ*) and random noises (*ε*):

1 $$ {IR}_{ij}^s=\mu \left({IR}_{ij}^s\right)+\xi \left({IR}_{ij}^s\right)+\varepsilon \left({IR}_{ij}^s\right). $$

We used the Fourier series to represent different oscillations of $\mu \left({IR}_{ij}^s\right)$ and $\xi \left({IR}_{ij}^s\right)$ in (1) by a linear combination of sinusoidal functions with different frequencies, magnitudes and phases [23]:

2 $$ \mu \left({IR}_{ij}^s\right)={\mu}_0^s+\sum \limits_{p=1}^P{A}_{\mu p}^s\sin 2\uppi \frac{k_p}{60}{t}_{ij}+\sum \limits_{p=1}^P{B}_{\mu p}^s\cos 2\uppi \frac{k_p}{60}{t}_{ij},{k}_p=1:P, $$

3 $$ \xi \left({IR}_{ij}^s\right)={\xi}_0^s+\sum \limits_{q=1}^Q{A}_{\xi q}^s\sin 2\uppi \frac{k_q}{12}{t}_{ij}+\sum \limits_{q=1}^Q{B}_{\xi q}^s\cos 2\uppi \frac{k_q}{12}{t}_{ij},{k}_q=1:Q. $$

Here P and Q are the number of the sine and cosine functions representing the trend and seasonal components, respectively, ${\mu}_0^s$ and ${\xi}_0^s$ are the intercepts defining the baselines of the trend and seasonal components, ${A}_{\mu p}^s$, ${B}_{\mu p}^s$, ${A}_{\xi q}^s$ and ${B}_{\xi q}^s$ are the Fourier coefficients and *t*_*ij*_ indexes time. The amplitude of the trend equals: ${C}_{\mu p}^s=\operatorname{sign}\left({B}_{\mu p}^s\right){\left({A_{\mu p}^s}^2+{B_{\mu p}^s}^2\right)}^{1/2}$; that of the seasonal component equals: ${C}_{\xi q}^s=\operatorname{sign}\left({B}_{\xi p}^s\right){\left({A_{\xi q}^s}^2+{B_{\xi q}^s}^2\right)}^{1/2}$, where sign is the function extracting the sign of a real number [24].

Iterative weighted least square estimation was used to fit (2) and (3) by minimizing the generalized cross-validation value: *GCV* = *RSS* + λ × *PEN*_*L*_, where *RSS* is the weighted residual sum of squares, *λ* is the smoothing parameter and *PEN*_*L*_ is a penalty term as a measure of Fourier series roughness [25].

### Identifying driving factors of seasonality

To contrast the effects of preschooling period as a proxy measure of the social contacts and the weather season on the seasonality of the disease, we further stratified the seasonal components $\xi \left({IR}_{ij}^s\right)$ into three temporal strata: T_1_ is the dry and preschooling season from January to May, T_2_ is the dry and summer holiday season from June to July and T_3_ is the rainy and preschooling season from August to December. Hence, for all *j* and *s*, $\xi \left({IR}_{ij}^s\right)$ belong to T_1_ for *i* = 1:5, to T_2_ for *i* = 6:7 and to T_3_ for *i* = 8:12, respectively.

To relate $\xi \left({IR}_{ij}^s\right)$ of T_1_, T_2_ and T_3_ to the potential risk factors, a spatial-temporal regression model was deployed:

4 $$ {\xi}_T\left({IR}_{ij}^s\right)={\beta}_{T0}^s+\sum \limits_{n=1}^N{\beta}_{Tn}^s{X}_{Tn ij}^s+{\beta}_{T\left(N+1\right)}^s{P}_{Ti}+{\upvarepsilon}_{Ti j}^s,T={\mathrm{T}}_1:{\mathrm{T}}_3, $$

where *N* is the total number of the weather covariates, ${\beta}_{T0}^s$ and ${\beta}_{Tn}^s$ are the regression parameters, ${X}_{Tnij}^s$ are the weather covariates, ${\upvarepsilon}_{Tij}^s$ are the spatial-temporal autoregressive regression residuals, *P*_*Ti*_ are the number of months at preschools, cumulated from August current year to May next year. Notice that *P*_*Ti*_ did not vary with *s* and *j* as they were obtained by: $$ {P}_{Ti}=\left\{\begin{array}{c}i+5\ \mathrm{for}\ i\in {\mathrm{T}}_1\\ {}0\ \mathrm{for}\ i\in {\mathrm{T}}_2\\ {}i-7\ \mathrm{for}\ i\in {\mathrm{T}}_3\ \end{array}\right. $$.

To account for spatial-temporal autocorrelation remaining in the residual, we applied the Spatial-Temporal AutoRegressive model (STAR) [26] for the error term ${\upvarepsilon}_{Tij}^s$ in (4):

5 $$ {\upvarepsilon}_{T ij}^s={\gamma}_{T1}^s{\upvarepsilon}_{T\left(\mathrm{i}+1\right)j}^s+{\gamma}_{T2}^s{\upvarepsilon}_{T\left(\mathrm{i}-1\right)j}^s+\sum \limits_{m=1}^M{\rho}_{T m}^s{\varepsilon}_{T ij m}^s+{\upomega}_{T ij}^s,\mathrm{with}\ {\upomega}_{T ij}^s\sim N\left(0,{\left({\sigma}_{T\omega}^s\right)}^2\right) $$

where *M* is the total number of the first-order spatial neighbors of S_*s*_, ${\gamma}_{T1}^s$ and ${\gamma}_{T2}^s$ are the temporal autoregressive coefficients, ${\rho}_{Tm}^s$ are the spatial autoregressive coefficients, ${\left({\sigma}_{T\omega}^s\right)}^2$ is the variance of the i.i.d. random error ${\upomega}_{Tij}^s$. The model parameters were estimated by maximum likelihood (ML) [27]. Schwarz’s Bayesian criterion (BIC) was used to indicate the fitness of the models. The Shapiro–Wilk test [28] was used for normality test. The analyses and modellings were executed in R. 3.4.4, using mainly stat4 library [29] and fda library [30].

## Results

### Seasonality at multiple district level

The seasonality of ${IR}_{ij}^s$ was evidenced by the variations of the corresponding ${SI}_i^s$ (Table 1). For all spatial strata, the maximum ${SI}_i^s$ > 100% fell into two periods, between April and May and between September and October. These indicate that, for example for S_2_ in April, the median incidence proportion was 1.66 times higher than the average incidence proportion of the year. The minimum ${SI}_i^s$ < 60% were in the period between December and January. These show that, for example for S_2_ in January, the median incidence proportion was 2.5 times lower than the average incidence proportion of the year.

**Table 1:** Seasonal indices of ${IR}_{ij}^s$ for 3 × 3 spatial-temporal strata in the mainland of Da Nang city

Strata		T_1_					T_2_		T_3_				
Month		Jan	Feb	Mar	Apr	May	Jun	Jul	Aug	Sep	Oct	Nov	Dec
SI % (Std. Error)	S_1_	44.6 (19.5)	52.5 (15.4)	123.0 (41.9)	138.6 (38.1)	106.9 (52.8)	83.0 (27.4)	108.1 (23.0)	105.0 (17.3)	136.9 (31.7)	158.1 (41.7)	88.1 (36.2)	55.1 (15.5)
	S_2_	41.0 (25.0)	72.9 (26.4)	94.6 (41.9)	165.7 (43.3)	107.0 (45.5)	73.5 (27.4)	121.7 (21.4)	105.0 (10.4)	138.1 (36.2)	136.3 (27.4)	89.7 (38.2)	54.7 (19.56)
	S_3_	49.4 (25.7)	67.1 (36.5)	122.1 (32.0)	142.5 (54.5)	137.8 (43.7)	86.5 (28.4)	93.8 (26.7)	124.4 (18.1)	131.6 (38.6)	121.8 (29.4)	70.7 (16.9)	52.3 (21.0)

**Fig. 2:**
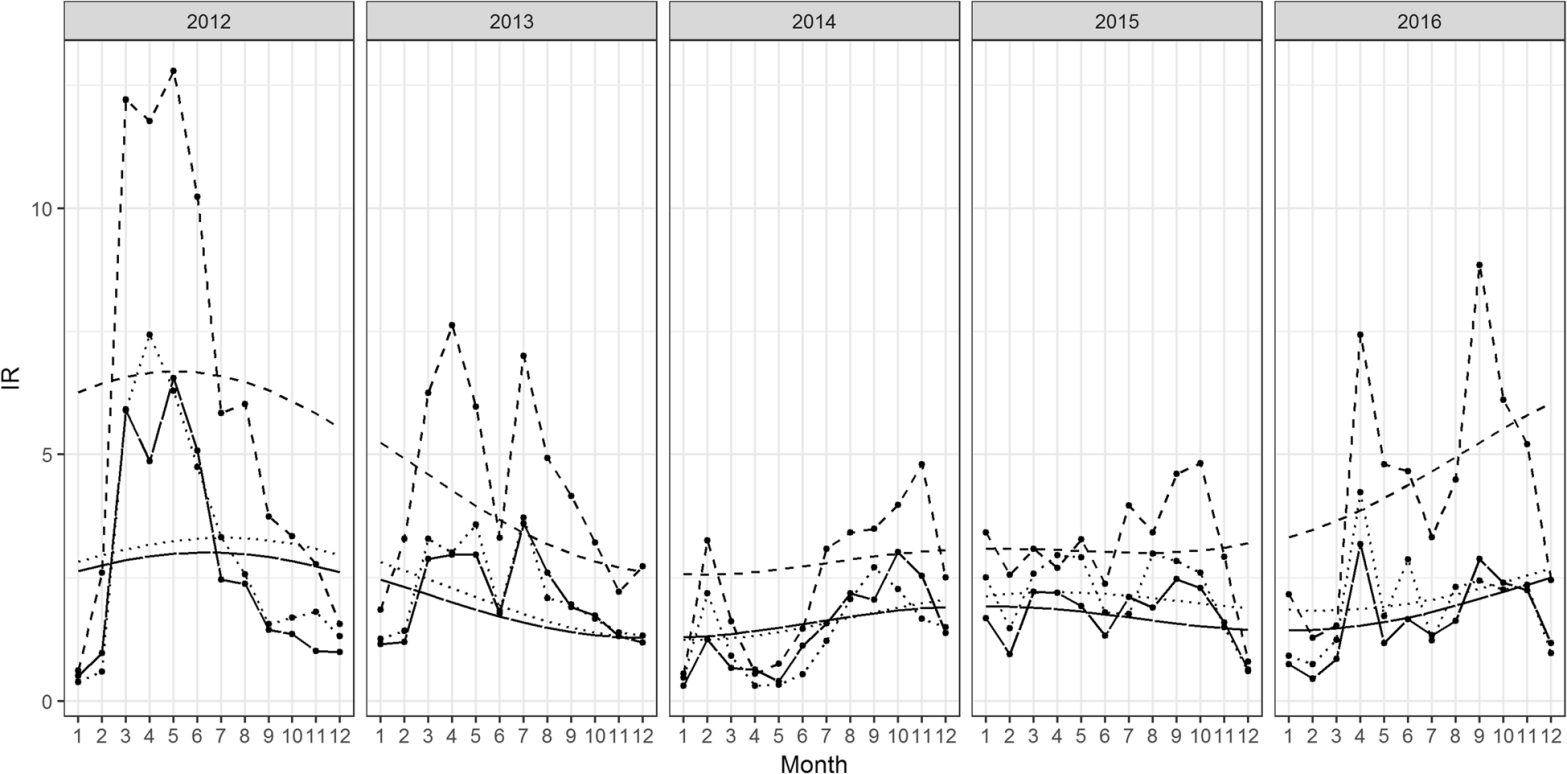
Time plot of ${IR}_{ij}^s$ and the temporal trends for S_1_ (long-dashed line), S_2_ (dashed line) and S_3_ (dotted line). ${IR}_{ij}^s$ in the dry season had larger magnitude than in the rainy season

As outcomes from the iterative fitting processes, the optimally fitted trends of S_1_ and S_2_ had two sine and two cosine functions (*P* = 2) and the optimal logλ = 4 corresponding to the minimum values of the *GCV* equal to 2.28 and 3.33, respectively. For S_3_, the fitted model included four of each of the trigonometric functions (*P =* 4) with logλ = 2 and the minimum *GCV* equal to 6.1. The fitted models for the seasonality (3) have five of each sine and cosine functions (*Q* = 5) with logλ = 0 and the minimum *GCV* equal to 0.87, 2.6 and 4.1 for the S_1_, S_2_ and S_3_, respectively. The trends show a gradual decrease from 2012 to 2015, whereas from the end of 2015 onwards, they increase (Fig. 2). The magnitude is highest in S_2_, being approximately double to those of S_1_ and S_3_.

**Fig. 3:**
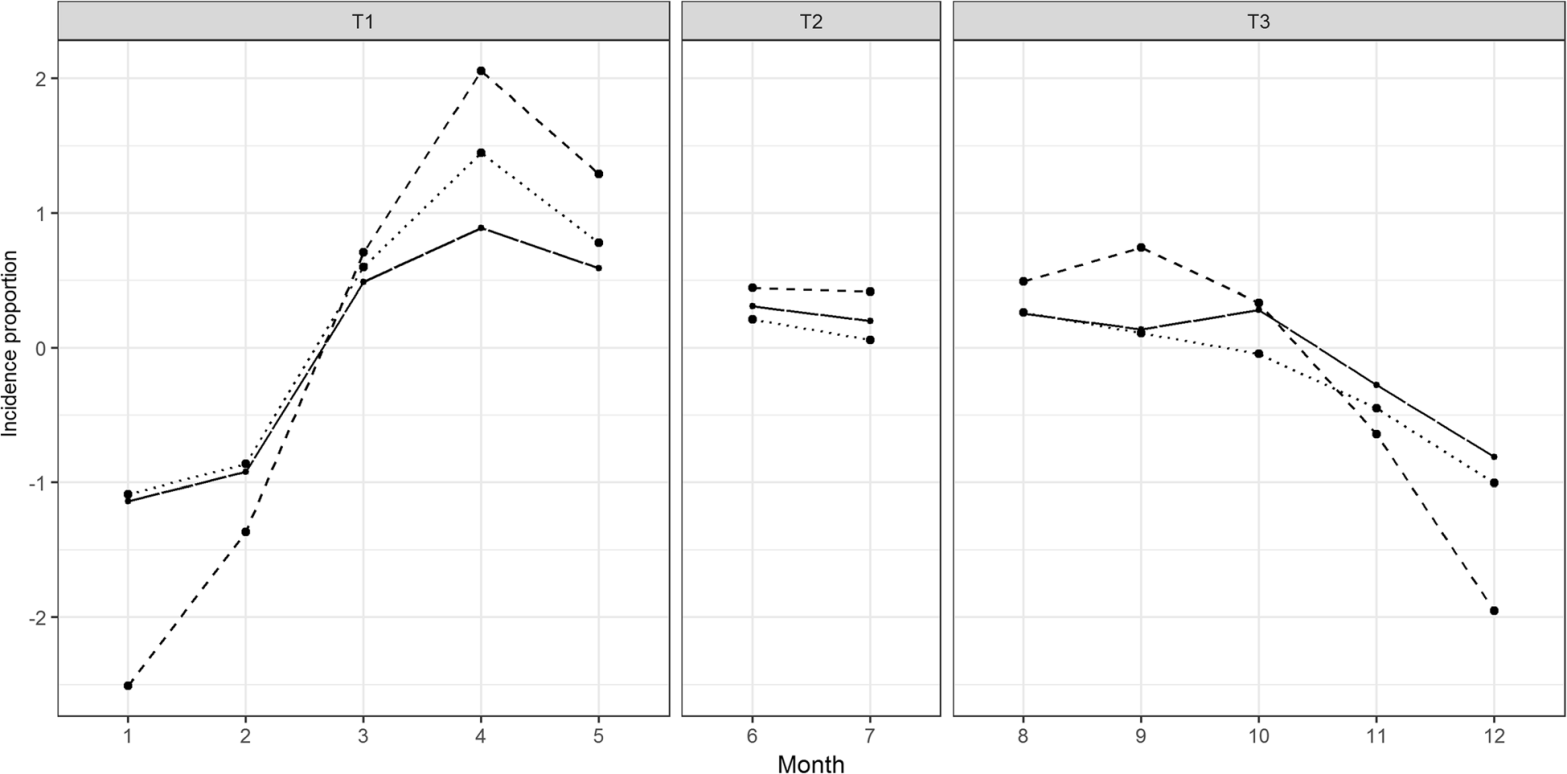
Seasonality of ${IR}_{ij}^s$ from the mainland of Da Nang city estimated from the 5 year time series for S_1_ (long-dashed line), S_2_ (dashed line) and S_3_ (dotted line). The highest peaks occurred in the middle of the dry season (April). The other peaks occurred after the children went to preschools

Fig 3 presents the seasonal patterns of ${IR}_{ij}^s$. In T_1_, all the peaks fell within April as indicated by the maximum ${SI}_i^s$ in Table 1. In T_3_, the peaks occurred in August (S_3_), September (S_2_) and October (S_1_). All the deepest troughs were in January, whereas the small troughs occurred in June. The maximum amplitude of seasonality was equal to 4.56 infected U5s per 1,000 U5s at risk per month.

### Driving factors of seasonality

**Fig. 4:**
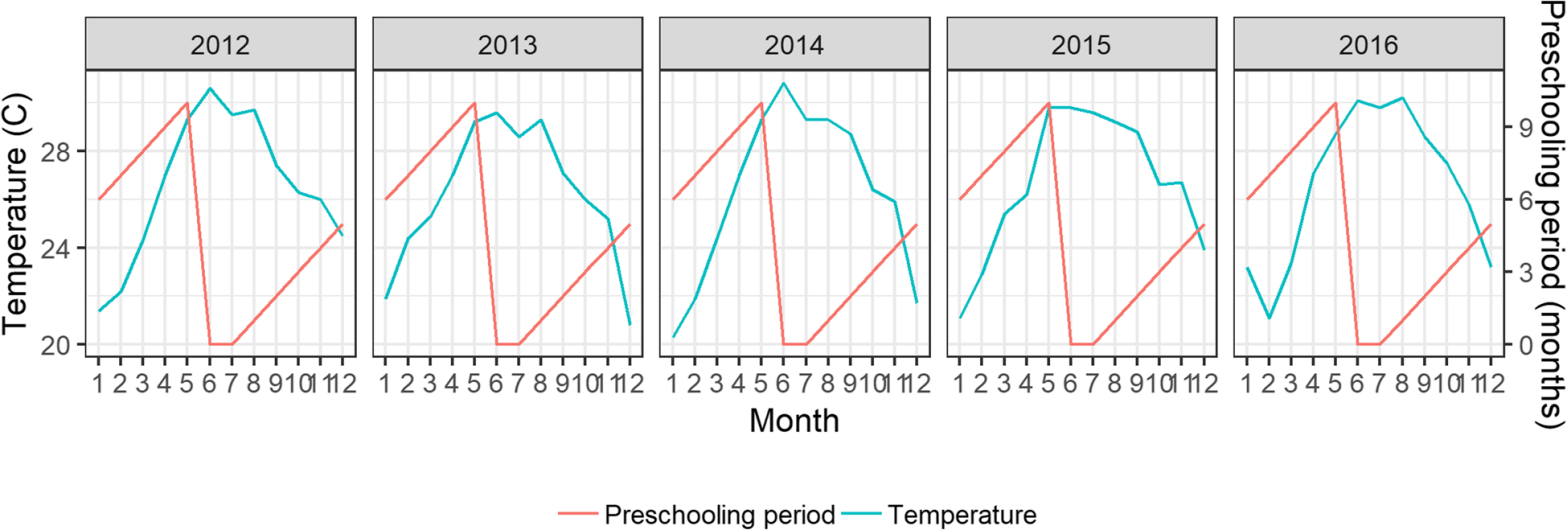
Temporal variation of the driving factors of the seasonality of HFMD incidences: the monthly average ambient temperature (cyan line) and the cumulative preschooling period (red line)

The monthly relative humidity and sunny hours in Da Nang city in the study period were significantly correlated at lag zero with the average temperature (*r*_*lag0*_ = − 0.82 and 0.84, respectively). Meanwhile, $\xi \left({IR}_{ij}^s\right)$ from all three spatial strata had significantly lower correlation with the rainfall than with the average temperature at lag zero (*r*_*lag0*_ = − 0.13, − 0.10, − 0.23 versus 0.74, 0.74, 0.63 for S_1_, S_2_, S_3_, respectively). The average temperature was apparently the sole explanatory weather variable included or *N* = 1 in (4). The maximum average temperature of over 30 °C occurred in May and June; the minimum of approximately 22 °C occurred in January every year (Fig. 4). The ${\xi}_T\left({IR}_{ij}^s\right)$ all satisfied the Shapiro-Wilk normality test (*p*-value < 0.05), thus satisfying the specifications of (4) and (5) without any need for transformation.

The results of the *q*-statistic show that the spatial-temporal stratification into nine strata reflecting the influences of the density of U5s and the monthly average temperature yielded a significant homogeneity within the strata. The *q*-statistic of the nine spatial-temporal strata was equal to 0.21 (*p*-value = 0.00), while that of the combinations of the months versus the districts was not significant, *q*-statistic = 0.26 (p-value = 0.24).

To compare the effects of the average temperature and the preschooling period (Fig 4), the parameters in (4) and (5) were consequently estimated for two sub-models: 1) with preschooling period or ${\beta}_{T\left(N+1\right)}^s={\beta}_{T2}^s$ and 2) without preschooling period or ${\beta}_{T\left(N+1\right)}^s=0$. Accordingly, every spatial-temporal stratum had two sets of estimated parameters, except for those in T_2_ because children did not attend preschools in this period. The estimated results are presented in Tables 2 and 3. The effects of the spatial-temporal autocorrelations were taken into account in these estimations, resulting in estimated parameters as presented in Tables 4 and 5 in Appendix. The corresponding BIC values of the selected models were all minimized.

**Table 2:** ML estimates of the spatial-temporal regression coefficients between the $\xi \left({IR}_{ij}^s\right)$ and the average temperature, *without* the cumulative preschooling period included

Strata	T_1_			T_2_			T_3_		
Parameter (Std. Error)	S_1_	S_2_	S_3_	S_1_	S_2_	S_3_	S_1_	S_2_	S_3_
${\boldsymbol{\beta}}_{\boldsymbol{T}\mathbf{0}}^{\boldsymbol{s}}$	5.44 (2.08)	7.12 (2.45)	4.10 (1.47)	3.25 (0.73)	8.83 (0.00)	6.55 (0.00)	1.57 (0.65)	−2.81 (0.08)	0.69 (0.35)
${\boldsymbol{\beta}}_{\boldsymbol{T}\mathbf{1}}^{\boldsymbol{s}}$ (Temperature)	−0.18 (0.07)	− 0.25 (0.09)	− 0.14 (0.05)	− 0.10 (0.02)	− 0.00 (0.00)	−0.74 (0.00)	− 0.12 (0.03)	0.02 (0.00)	− 0.08 (0.01)
BIC	−23.28	−9.63	−45.38	−15.73	− 95.88	−95.23	−41.90	−69.34	− 66.72

**Table 3:** ML estimates of the spatial-temporal regression coefficients between the $\xi \left({IR}_{ij}^s\right)$ and the average temperature *including* the cumulative preschooling period

Strata	T_1_			T_3_		
Parameters (Std. Error)	S_1_	S_2_	S_3_	S_1_	S_2_	S_3_
${\boldsymbol{\beta}}_{\boldsymbol{T}\mathbf{0}}^{\boldsymbol{s}}$	−5.16 (0.61)	8.00 (3.73)	−5.57 (0.55)	0.8 (0.03)	0.64 (0.03)	0.65 (0.09)
${\boldsymbol{\beta}}_{\boldsymbol{T}\mathbf{1}}^{\boldsymbol{s}}$ (Average temperature)	0.08 (0.03)	0.03 (0.04)	0.07 (0.02)	−0.00 (0.00)	−0.00 (0.00)	− 0.01 (0.00)
${\boldsymbol{\beta}}_{\boldsymbol{T}\mathbf{2}}^{\boldsymbol{s}}$ (Preschooling period)	0.32 (0.05)	−1.25 (0.53)	0.39 (0.05)	−0.40 (0.01)	−0.29 (0.00)	− 0.25 (0.01)
BIC	−50.00	− 68.79	−57.86	− 189.86	− 98.63	− 166.29

T_2_ is the transitional period between the dry and rainy season and also the period of summer holidays. Thus, the interest of studying the driving factor of the seasonality laid in T_1_ and T_3_. Table 2 shows that in the sub-model one, the average temperature was negatively correlated with $\xi \left({IR}_{ij}^s\right)$ in T_1_. This negative ${\beta}_{T1}^s$ indicates a mismatch between the peak of the seasonality of the disease incidences and the average temperature. In sub-model two as can be seen in Table 3, the ${\beta}_{T2}^s$ had negative values for all strata in T_3_; whereas, the ${\beta}_{T1}^s$ were minimal. The average temperature therefore had no significant effect in T_3_.

## Discussion

In this section, we discuss our main findings, the consistency of the seasonal patterns extracted from ${IR}_{ij}^s$ using our methods compared to other methods and we also highlight the limitations of this study.

In this study, we have decomposed the monthly time series of the adjusted incidence proportions of childhood HFMD infections for the spatial-temporal strata of the mainland of Da Nang city to reveal the trend and the seasonality. The yearly larger outbreaks simultaneously happened in all three spatial strata in April. The variation of the seasonality of the second smaller outbreaks in T_3_ is intriguing. This variation suggests the effects of the season of increasing social contacts among U5s when they go to preschool on the onset of HFMD infection.

We have shown statistical evidences of these effects during the preschooling period. The positive values of the spatial-temporal regression coefficients imply the simultaneity of the peaks and vice versa [21]. The mismatch between the maximum incidence proportions and the maximum average temperature indicates that the increase of the average temperature was the driving factor, not its maximum values. In the last two quarters of all years, the average temperature was not the predominant risk factor. The contrary was evident in T_1_. At small areas of multiple districts in the study area, the increasing social contact has been shown to be the important driving factor of the geographical variation of the seasonality of the childhood HFMD outbreaks.

The estimated spatial-temporal regression parameters of (4) in T_3_ are interestingly more informative than those in T_1_ because all the smaller peaks occurred in T_3_. As the period at the preschools in T_3_ cumulatively increased from August to December, the negative values of ${\beta}_{T2}^s$ indicate the occurrence of the smaller peaks at the beginning of the preschooling period. Recall from the study of Horby et al. [17] that the physical contacts of U5s are most intense within this age group in Viet Nam. Given that the physical contact is the prominent means of passing HFMD viruses, the nature, frequency and duration of the contacts of those children during the preschooling period explain the occurrence and the geographical dynamics of these smaller outbreaks.

The trends show the occurrences of the large outbreaks at the beginning and at the end of the observed period. Study of a longer period of time would show a long-term oscillation of the large outbreaks with the frequency of every two to 3 years in this city. The possible reasons can be the required lapse of time to cumulate the critical susceptible population [31] or the existence of multiple viral strains in the study area [32, 33].

The decomposed trends and seasonal components were highly correlated with those fitted by the method of seasonal and trend decomposition using Loess [34]. The correlation coefficients of the trends from both methods at lag zero varied from 0.85 to 0.90. Those of the seasonality varied from 0.83 to 0.97. The similar results from both decomposition methods imply the consistency of the seasonality derived from the Fourier decomposition method. This method provides a semi-parametric approach to extract different components of a time series including the long-term and short-term irregular and regular fluctuations, taking into account effects of possible confounders [35, 36]. Using this approach instead of the common seasonal ARIMA, the drawbacks of stationarization by differencing and statistical transformation can be avoided [37]. Moreover, Fourier decomposition in combination with the spatial-temporal autoregressive model allow both spatial and temporal autocorrelation existing in the data to be included into the model calibration. This reduces the biases in the estimation of the associations between the incidence proportions and the risk factors. Notwithstanding, from a statistical point of view, the small number of spatial strata in the study area places one of the limitations of the study to include the effects of the spatial auto-correlation. In addition, the separation the temporal trend from the time series was based mainly upon expert judgments. In other words, the efficiency of the Fourier decomposition in many cases relies on the expert’s familiarity with the understudied phenomena.

## Conclusions

HFMD has been becoming one of the most important pediatric NTDs in the Northeastern and Southeastern Asian countries. By applying spatial-temporal statistical analyses, the results have shown that at multiple district level, the social contact is the important driving factor of the spatial variation of the disease’s outbreaks. This study provides statistical evidences of the effects of the seasonality of increasing social contact amongst U5s on geographical dynamics of HFMD infection outbreaks. Our findings contribute to extend the understanding of the underlying driving factors of the disease dynamics at small areas. This contribution is necessary to inform the next insightful research into the spatial-temporal dynamics of the transmission of the NTDs within the local population.

## Data Availability

The dataset of HFMD incidences in Da Nang city analyzed during the current study is publicly available on the website of Da Nang city Preventive medicine center https://ksbtdanang.vn/. The dataset of the monthly average ambient temperature in Da Nang city is publicly available on the website of Viet Nam institute of meteorology, hydrology and climate change http://www.imh.ac.vn. The R code of the analyses and models are available from the corresponding author on reasonable request.

## Appendix

**Table 4:** ML estimates of the spatial and temporal auto-regression coefficients of the sub-model one

Strata	T_1_			T_2_			T_3_		
Parameters (Std. Error)	S_1_	S_2_	S_3_	S_1_	S_2_	S_3_	S_1_	S_2_	S_3_
${\boldsymbol{\gamma}}_{\boldsymbol{T}\mathbf{1}}^{\boldsymbol{s}}$	0.04 (0.06)	0.08 (0.05)	0.23 (0.04)	−0.37 0.70	− 0.17 (0.00)	0.00 (0.00)	0.07 (0.06)	2.26 (0.06)	0.22 (0.05)
${\boldsymbol{\gamma}}_{\boldsymbol{T}\mathbf{2}}^{\boldsymbol{s}}$	0.42 (0.14)	0.38 (0.05)	−0.09 (0.02)	0.26 0.28	−0.19 (0.00)	1.50 (0.02)	−0.55 (0.12)	0.87 (0.02)	0.38 (0.03)
${\boldsymbol{\rho}}_{\boldsymbol{T}\mathbf{1}}^{\boldsymbol{s}}$	−0.61 (0.18)	−0.61 (0.21)	1.03 (0.04)	1.70 (0.00)	1.05 (0.00)	−0.51 (0.02)	0.16 (0.05)	−1.26 (0.11)	0.35 (0.07)
${\boldsymbol{\rho}}_{\boldsymbol{T}\mathbf{2}}^{\boldsymbol{s}}$	1.54 (0.21)	1.62 (0.18)	0.09 (0.02)	0.18 (0.00)	0.27 (0.00)	0.00 (0.00)	1.28 (0.18)	−1.05 (0.10)	0.06 (0.02)
${\boldsymbol{\sigma}}_{\boldsymbol{T}\boldsymbol{\omega}}^{\boldsymbol{s}}$	0.09 (0.01)	0.12 (0.02)	0.04 (0.01)	0.04 (0.00)	0.00 (0.00)	0.00 (0.00)	0.06 (0.01)	0.04 (0.01)	0.04 (0.01)

**Table 5:** ML estimates of the spatial and temporal auto-regression coefficients of the sub-model two

Strata	T_1_			T_3_		
Parameter (Std. Error)	S_1_	S_2_	S_3_	S_1_	S_2_	S_3_
${\boldsymbol{\gamma}}_{\boldsymbol{T}\mathbf{1}}^{\boldsymbol{s}}$	−0.25 (0.04)	0.15 (0.03)	1.61 (0.06)	−0.51 (0.01)	−1.68 (0.04)	−0.06 (0.01)
${\boldsymbol{\gamma}}_{\boldsymbol{T}\mathbf{2}}^{\boldsymbol{s}}$	0.07 (0.02)	0.04 (0.01)	8.12 (0.02)	−0.04 (0.02)	0.04 (0.01)	−0.21 (0.01)
${\boldsymbol{\rho}}_{\boldsymbol{T}\mathbf{1}}^{\boldsymbol{s}}$	−0.07 (0.03)	0.21 (0.09)	5.82 (0.01)	−0.24 (0.02)	−0.71 (0.07)	0.23 (0.01)
${\boldsymbol{\rho}}_{\boldsymbol{T}\mathbf{2}}^{\boldsymbol{s}}$	0.96 (0.04)	0.84 (0.09)	6.62 (0.00)	2.79 (0.06)	8.84 (0.01)	0.21 (0.00)
${\boldsymbol{\sigma}}_{\boldsymbol{T}\boldsymbol{\omega}}^{\boldsymbol{s}}$	0.05 (0.01)	0.035 (0.01)	0.06 (0.01)	0.00 (0.00)	0.02 (0.00)	0.01 (0.00)

## Abbreviations

BIC: Schwarz’s Bayesian criterion
CA16: *Coxsackievirus A16*
EV71: Human *Enterovirus 71*
HFMD: Hand-foot-mouth disease
ML: Maximum likelihood
NTDs: Neglected tropical diseases
S: Spatial stratum
SI: Seasonal index
STS: Spatial-temporal statistical methods
T: Temporal stratum
U5s: Children under 5 years of age
